# Supplementary material for: Hot spot prediction in protein-protein interactions by an ensemble system
Source: BMC Syst Biol. 2018 Dec 31;12(Suppl 9):132. doi: 10.1186/s12918-018-0665-8 (PMC6311905; doi:10.1186/s12918-018-0665-8)
Supplement: Supplementary file 2 — All relevant MATLAB code. A simple Matlab implement of our predictor. (ZIP 19,742 kb) [file 12918_2018_665_MOESM2_ESM.zip › Additional file 2/Read ASA/ASA website/BID/1ES7A.htm]

 NetSurfP - Protein Surface Accessibility and Secondary Structure Predictions

  

|  |  |  |
| --- | --- | --- |
|  |  | NetSurfP - Protein Surface Accessibility and Secondary Structure Predictions  Technical University of Denmark |

  


---

```
# For publication of results, please cite:
# A generic method for assignment of reliability scores applied to solvent accessibility predictions.
# Bent Petersen, Thomas Nordahl Petersen, Pernille Andersen, Morten Nielsen and Claus Lundegaard
# BMC Structural Biology 2009, 9:51 doi:10.1186/1472-6807-9-51
#
# Column 1: Class assignment - B for buried or E for Exposed - Threshold: 25% exposure, but not based on RSA
# Column 2: Amino acid
# Column 3: Sequence name
# Column 4: Amino acid number
# Column 5: Relative Surface Accessibility - RSA
# Column 6: Absolute Surface Accessibility
# Column 7: Z-fit score for RSA prediction
# Column 8: Probability for Alpha-Helix
# Column 9: Probability for Beta-strand
# Column 10: Probability for Coil
E M  1ES7A                  1    0.742 148.414  -1.896   0.003   0.003   0.994
E A  1ES7A                  2    0.660  72.776   0.085   0.058   0.017   0.925
E Q  1ES7A                  3    0.629 112.304   0.513   0.115   0.016   0.868
E A  1ES7A                  4    0.534  58.825  -0.708   0.181   0.016   0.803
E K  1ES7A                  5    0.627 128.933   0.605   0.184   0.043   0.773
E H  1ES7A                  6    0.467  84.911   0.472   0.268   0.043   0.689
E K  1ES7A                  7    0.607 124.839   0.626   0.191   0.086   0.723
E Q  1ES7A                  8    0.460  82.138   1.023   0.191   0.086   0.723
E R  1ES7A                  9    0.473 108.409   0.058   0.184   0.043   0.773
E K  1ES7A                 10    0.634 130.434   0.021   0.113   0.043   0.844
E R  1ES7A                 11    0.671 153.682   0.340   0.058   0.017   0.925
E L  1ES7A                 12    0.645 118.136   0.059   0.016   0.005   0.979
E K  1ES7A                 13    0.667 137.120   0.805   0.018   0.019   0.964
E S  1ES7A                 14    0.597  69.992   0.304   0.018   0.019   0.964
E S  1ES7A                 15    0.397  46.564   1.874   0.004   0.085   0.910
B C  1ES7A                 16    0.019   2.696  -0.242   0.004   0.420   0.576
E K  1ES7A                 17    0.305  62.697   0.799   0.003   0.718   0.279
E R  1ES7A                 18    0.360  82.509  -0.122   0.002   0.816   0.182
E H  1ES7A                 19    0.292  53.206   1.220   0.002   0.816   0.182
E P  1ES7A                 20    0.540  76.697   0.649   0.002   0.816   0.182
B L  1ES7A                 21    0.088  16.168   0.643   0.002   0.816   0.182
E Y  1ES7A                 22    0.420  89.754   0.748   0.001   0.900   0.099
B V  1ES7A                 23    0.033   5.011   0.203   0.002   0.816   0.182
E D  1ES7A                 24    0.362  52.107   1.296   0.021   0.756   0.223
B F  1ES7A                 25    0.081  16.196   0.270   0.022   0.552   0.426
E S  1ES7A                 26    0.555  64.999   1.275   0.022   0.552   0.426
E D  1ES7A                 27    0.514  74.010   0.382   0.022   0.552   0.426
B V  1ES7A                 28    0.147  22.563   1.066   0.022   0.359   0.619
E G  1ES7A                 29    0.437  34.400  -0.828   0.020   0.205   0.775
B W  1ES7A                 30    0.258  62.145  -0.295   0.019   0.141   0.840
E N  1ES7A                 31    0.512  74.928  -0.989   0.020   0.205   0.775
E D  1ES7A                 32    0.359  51.732  -0.084   0.021   0.279   0.699
B W  1ES7A                 33    0.102  24.555   0.565   0.023   0.655   0.322
B I  1ES7A                 34    0.045   8.362   0.438   0.021   0.756   0.223
B V  1ES7A                 35    0.079  12.127  -0.133   0.021   0.756   0.223
B A  1ES7A                 36    0.150  16.530   0.891   0.021   0.451   0.528
B P  1ES7A                 37    0.096  13.637  -0.188   0.019   0.141   0.840
E P  1ES7A                 38    0.549  77.903   0.065   0.018   0.047   0.935
E G  1ES7A                 39    0.439  34.526  -0.724   0.018   0.088   0.893
B Y  1ES7A                 40    0.104  22.289   0.135   0.021   0.279   0.699
E H  1ES7A                 41    0.336  61.064   0.369   0.023   0.655   0.322
B A  1ES7A                 42    0.063   6.932  -0.554   0.021   0.756   0.223
B F  1ES7A                 43    0.119  23.863  -0.436   0.018   0.846   0.136
B Y  1ES7A                 44    0.239  51.032   0.298   0.018   0.846   0.136
B C  1ES7A                 45    0.020   2.766  -0.271   0.021   0.756   0.223
E H  1ES7A                 46    0.341  62.064   0.056   0.022   0.552   0.426
B G  1ES7A                 47    0.079   6.194  -2.289   0.021   0.451   0.528
E E  1ES7A                 48    0.515  90.040   0.236   0.022   0.552   0.426
B C  1ES7A                 49    0.077  10.811   0.232   0.022   0.359   0.619
E P  1ES7A                 50    0.470  66.736  -1.189   0.064   0.216   0.721
B F  1ES7A                 51    0.240  48.228  -0.133   0.052   0.084   0.864
E P  1ES7A                 52    0.300  42.527  -0.941   0.053   0.043   0.903
B L  1ES7A                 53    0.200  36.712  -0.235   0.113   0.043   0.844
E A  1ES7A                 54    0.394  43.463  -1.639   0.115   0.016   0.868
E D  1ES7A                 55    0.575  82.829  -1.855   0.058   0.017   0.925
E H  1ES7A                 56    0.394  71.760  -1.362   0.053   0.043   0.903
B L  1ES7A                 57    0.167  30.523  -1.238   0.052   0.084   0.864
E N  1ES7A                 58    0.299  43.832  -0.873   0.018   0.047   0.935
B S  1ES7A                 59    0.179  21.026  -2.280   0.058   0.017   0.925
B T  1ES7A                 60    0.174  24.134  -1.685   0.257   0.016   0.727
B N  1ES7A                 61    0.138  20.262  -1.085   0.622   0.015   0.363
B H  1ES7A                 62    0.107  19.409  -0.965   0.802   0.014   0.185
B A  1ES7A                 63    0.115  12.629  -1.527   0.938   0.007   0.055
B I  1ES7A                 64    0.084  15.522   0.037   0.950   0.028   0.022
B V  1ES7A                 65    0.019   2.997   0.894   0.911   0.033   0.057
B Q  1ES7A                 66    0.128  22.807   0.724   0.911   0.033   0.057
B T  1ES7A                 67    0.173  23.981  -0.534   0.911   0.033   0.057
B L  1ES7A                 68    0.081  14.849   0.491   0.950   0.028   0.022
B V  1ES7A                 69    0.083  12.711   0.164   0.879   0.010   0.111
E N  1ES7A                 70    0.376  55.032   0.229   0.879   0.010   0.111
E S  1ES7A                 71    0.392  45.919  -0.435   0.717   0.014   0.269
E V  1ES7A                 72    0.368  56.577  -0.672   0.321   0.003   0.675
E N  1ES7A                 73    0.416  60.858  -0.697   0.053   0.005   0.942
E S  1ES7A                 74    0.548  64.261  -2.361   0.016   0.005   0.979
E K  1ES7A                 75    0.749 153.987  -1.056   0.003   0.003   0.994
E I  1ES7A                 76    0.302  55.907  -0.509   0.005   0.015   0.979
E P  1ES7A                 77    0.447  63.387  -0.887   0.005   0.015   0.979
E K  1ES7A                 78    0.457  94.087   0.149   0.005   0.015   0.979
B A  1ES7A                 79    0.177  19.461  -0.715   0.005   0.045   0.951
B C  1ES7A                 80    0.069   9.631  -0.303   0.005   0.262   0.733
B C  1ES7A                 81    0.035   4.956  -0.787   0.022   0.552   0.426
B V  1ES7A                 82    0.182  28.004   1.089   0.021   0.451   0.528
B P  1ES7A                 83    0.118  16.773  -0.249   0.021   0.279   0.699
E T  1ES7A                 84    0.360  49.960   0.253   0.021   0.279   0.699
E E  1ES7A                 85    0.496  86.581   0.728   0.021   0.279   0.699
B L  1ES7A                 86    0.215  39.403  -0.053   0.021   0.279   0.699
E S  1ES7A                 87    0.473  55.471  -0.360   0.004   0.085   0.910
E A  1ES7A                 88    0.351  38.658  -1.044   0.004   0.197   0.799
B I  1ES7A                 89    0.067  12.358   0.319   0.004   0.616   0.381
B S  1ES7A                 90    0.188  22.069  -0.069   0.002   0.816   0.182
B M  1ES7A                 91    0.038   7.524   0.369   0.001   0.900   0.099
B L  1ES7A                 92    0.047   8.569   0.428   0.001   0.959   0.040
B Y  1ES7A                 93    0.080  17.032   0.439   0.001   0.959   0.040
B L  1ES7A                 94    0.113  20.672   0.343   0.001   0.959   0.040
B D  1ES7A                 95    0.197  28.330   0.429   0.003   0.718   0.279
E E  1ES7A                 96    0.696 121.556  -0.071   0.004   0.138   0.858
E N  1ES7A                 97    0.775 113.401  -0.826   0.003   0.003   0.994
E E  1ES7A                 98    0.489  85.341  -0.179   0.005   0.015   0.979
E K  1ES7A                 99    0.398  81.786   0.891   0.004   0.616   0.381
B V  1ES7A                100    0.138  21.164   1.499   0.001   0.900   0.099
E V  1ES7A                101    0.326  50.168   1.584   0.001   0.959   0.040
B L  1ES7A                102    0.227  41.564   1.012   0.001   0.959   0.040
E K  1ES7A                103    0.345  71.028   1.519   0.001   0.959   0.040
E N  1ES7A                104    0.480  70.213   1.489   0.003   0.718   0.279
B Y  1ES7A                105    0.136  29.170   1.195   0.020   0.205   0.775
E Q  1ES7A                106    0.742 132.593   0.973   0.018   0.019   0.964
E D  1ES7A                107    0.722 104.040   0.941   0.018   0.019   0.964
B M  1ES7A                108    0.076  15.128   0.973   0.021   0.279   0.699
E V  1ES7A                109    0.323  49.707   1.746   0.018   0.846   0.136
B V  1ES7A                110    0.124  19.105   0.285   0.018   0.846   0.136
E E  1ES7A                111    0.530  92.556   1.096   0.021   0.756   0.223
E G  1ES7A                112    0.571  44.938   1.132   0.022   0.552   0.426
B C  1ES7A                113    0.098  13.745  -0.791   0.022   0.359   0.619
E G  1ES7A                114    0.425  33.487  -0.229   0.021   0.279   0.699
B C  1ES7A                115    0.188  26.353  -1.561   0.004   0.138   0.858
E R  1ES7A                116    0.687 157.254   0.670   0.005   0.015   0.979

Explain the output.  Go back.
```

---
